# Non-thermal argon plasma therapy alleviates atopic dermatitis by reducing keratinocyte oxidative stress

**DOI:** 10.1186/s43556-026-00641-y

**Published:** 2026-11-02

**Authors:** Young Joon Park, Mei Huang, Yun Sang Lee, Han-Seul Kim, Hee Young Kang, Chul-Ho Kim

**Affiliations:** 1 Department of Dermatology, Ajou University School of Medicine, 164 WorldCup-Ro, Yeongtong-Gu, Suwon, 16499 Korea; 2 Department of Molecular Science and Technology, Ajou University School of Medicine, Suwon, Korea; 3 Department of Otolaryngology, Ajou University School of Medicine, 164 WorldCup-Ro, Yeongtong-Gu, Suwon, 16499 Korea; 4 Department of Medical Sciences, Graduate School of Ajou University, Suwon, Korea

**Keywords:** Non-thermal plasma, Atopic dermatitis, Microbiome, IL-33, Oxidative stress, NRF2

## Abstract

Atopic dermatitis (AD) is a prevalent inflammatory skin disorder associated with a considerable disease burden. Despite the advent of biologics a substantial unmet need remains for effective treatment. We aimed to evaluate the effects of non-thermal argon plasma (NAP) in patients with mild-to-moderate AD. The Eczema Area Severity Index (EASI), Numeric Rating Scale (NRS), and Dermatology Life Quality Index (DLQI) were evaluated following AD treatment. 16 S rRNA sequencing was used to assess alterations in the skin microbiome. A mouse model of house dust mite (HDM)-induced AD was used to evaluate the effects of NAP on AD-like lesions, immune cell recruitment, and cytokine expression. Oxidative stress parameters and related molecular markers were analyzed after NAP treatment. NAP treatment led to a significant reduction in EASI, NRS, and DLQI scores compared with the sham group, reflecting an alleviation of AD symptoms and an enhancement in the quality of life. Microbiome analysis revealed significant alterations in the microbial diversity after NAP treatment. NAP ameliorated HDM-induced AD in mice, showing a decrease in epidermal thickness, immune cell recruitment, and the expression of key cytokines, particularly interleukin (IL)− 33, in keratinocytes. Furthermore, NAP treatment downregulated oxidative stress markers, corroborating the reduced IL-33 expression and underscoring the role of oxidative stress in AD pathology. Our study suggests the therapeutic potential of nonthermal plasma in the management of mild-to-moderate AD. Further extensive clinical trials are warranted to validate these findings and understand their long-term effects and implications.

**Trial registration**: CRIS, KCT0008949, retrospectively registered on October 10, 2023, at https://cris.nih.go.kr/cris/search/detailSearch.do?seq=25713.

**Supplementary Information:**

The online version contains supplementary material available at https://doi.org/10.1186/s43556-026-00641-y.

## Introduction

Atopic dermatitis (AD) is a chronic, relapsing inflammatory skin disorder. A recent comprehensive systematic analysis estimated the global prevalence of AD at 2.6%, corresponding to approximately 204 million affected individuals, including 103 million children and 101 million adults [1]. The Global Burden of Disease Study 2021 reported that the number of AD cases has increased over the past three decades and is projected to rise further through 2050 [2]. AD also contributes substantially to nonfatal disease burden worldwide, as measured by years lived with disability [2].

Significant advances in AD treatment have been achieved with the introduction of biological therapies [3]. The T helper 2 (Th2) response can be modulated using monoclonal antibodies targeting interleukin (IL)-4, with substantial clinical efficacy in AD [4]. Subsequently, additional human monoclonal antibodies targeting cytokines other than IL-4, such as IL-13, were developed and evaluated for AD treatment [5]. Despite their advantages, biologic therapies have several limitations. First, although these treatments demonstrate significant clinical efficacy, some patients continue to experience residual symptoms [6, 7]. Second, a subset of patients experience disease flares or substantial disease burden during therapy [8]. In addition, biologic therapies are associated with a considerable financial burden, and many patients require prolonged treatment because relapses are common after treatment withdrawal. Third, the need for injections may cause treatment-related anxiety in some patients. Taken together, biologic therapies are generally reserved for patients with uncontrolled moderate-to-severe AD, leaving those with mild-to-moderate disease with limited therapeutic options [3, 4].

Physical plasma consists primarily of charged particles, including electrons and ions, generated when a gaseous medium is exposed to an external energy source [9]. A key characteristic of plasma is its thermal state, which can range from high-temperature to non-thermal conditions. Nonthermal plasma (NTP), also referred to as cold atmospheric plasma (CAP), has been investigated in dermatology since the early 2000 s. NTP has been explored for applications including the treatment of skin infections, wound healing, and tissue regeneration [10]. Preclinical studies using mouse models of AD have reported that NTP modulates immune responses to reduce atopic inducers and can ameliorate AD-like skin inflammation [11, 12]. However, clinical evidence regarding the use of NTP devices for chronic inflammatory skin disorders remains limited. Non-thermal argon plasma (NAP), a subtype of NTP that uses argon as the working gas, has attracted increasing interest because of its favorable safety profile and potential dermatological applications. Although NAP has shown promising biological effects in experimental studies, its clinical efficacy and underlying mechanisms in patients with AD remain largely unexplored.

Therefore, we conducted an exploratory study to evaluate the efficacy and safety of NAP in patients with mild-to-moderate AD. We focused on the modulation of keratinocyte oxidative stress (OS) and IL-33 signaling as potential mechanisms underlying the therapeutic effects of NAP in AD. Based on our clinical and experimental findings, we propose that NAP may represent a novel and safe therapeutic option for patients with mild-to-moderate AD.

## Results

### NAP treatment alleviated acute eczematous lesions in mild-to-moderate AD

**Fig. 1:**
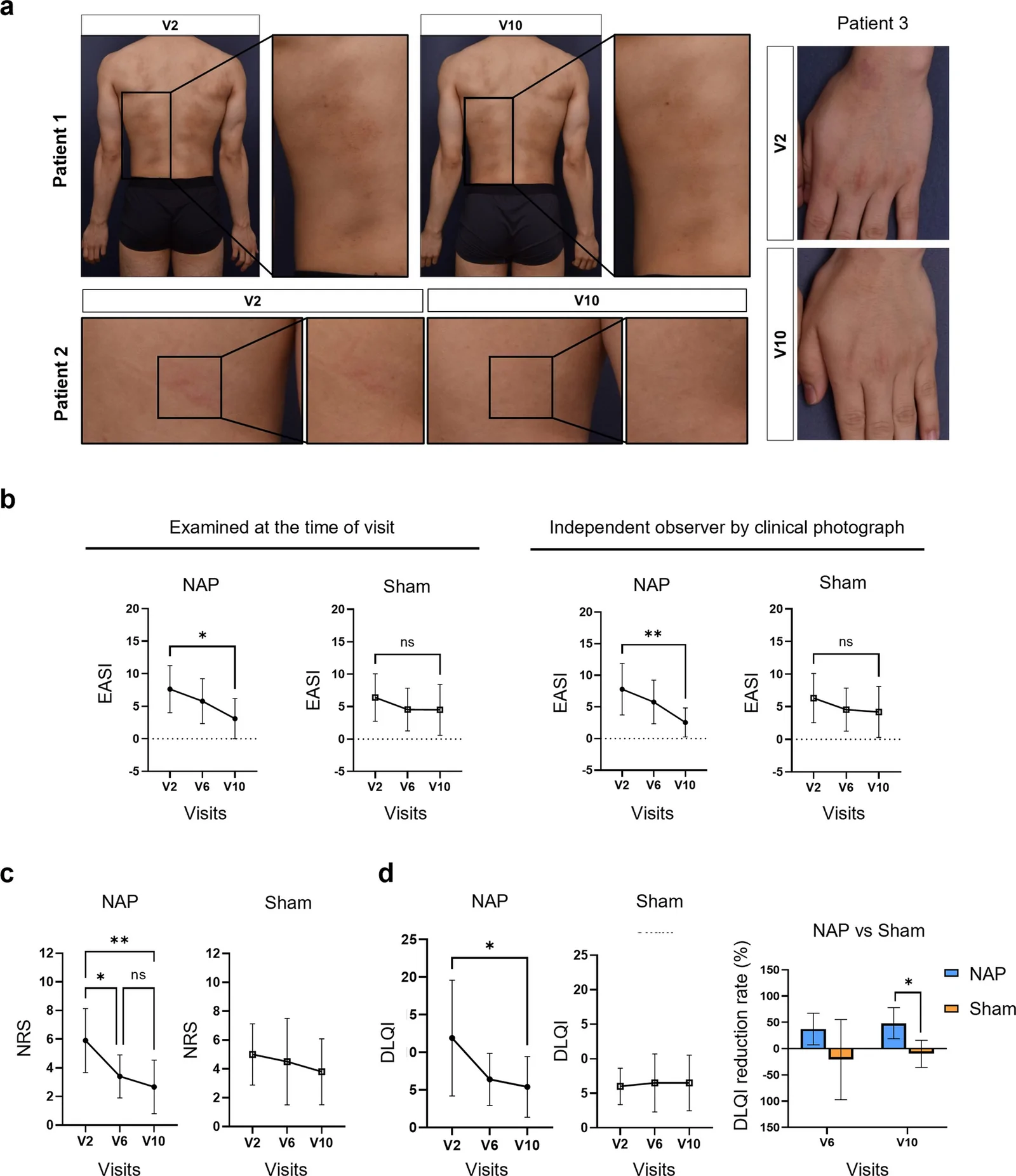
Short NAP treatment was sufficient to relieve mild-to-moderate AD symptoms. **a** Representative clinical photographs of patients with AD treated for 1 min. **b** EASI scores evaluated at the study visit by the independent observer using clinical photographs. **c**, **d** Changes in pruritus NRS and DLQI scores in the NAP-treated (*n* = 10) and sham device-treated groups (*n* = 5). Data are presented as mean ± SEM. *P < 0.05; ** *P* < 0.01. NAP, non-thermal argon plasma; NRS, Numeric Rating Scale; DLQI, Dermatology Life Quality Index; EASI, Eczema Area Severity Index; AD, atopic dermatitis

To evaluate NAP efficacy, 15 participants were enrolled, of whom 10 received active NAP treatment and 5 received sham treatment. An overview of the clinical study design and the NAP device is provided in Fig. S1. Baseline demographic and clinical characteristics are summarized in Table S1. Clinical efficacy was assessed at week 4 (V10) relative to the baseline (V2). Eczematous lesions showed visible clinical improvement after NAP treatment (Fig. 1a). Comparison of V2 and V10 showed a significant decrease in the EASI score in the NAP-treated group, as assessed both by a blinded observer at the study visit and by an independent observer using clinical photographs, in contrast to the sham device-treated group (Fig. 1b). NRS and DLQI scores were also significantly decreased in NAP-treated patients but not in sham-treated patients (Fig. 1c, d). Our findings indicate that NAP treatment for 1 min was sufficient to substantially alleviate AD symptoms, reduce pruritus, and improve quality of life. No significant changes in transepidermal water loss (TEWL) or total immunoglobulin E (IgE) were observed (Fig. S2a, b). Because total IgE reflects systemic atopic sensitization, it is unlikely to be altered by short-term, localized treatment. Similarly, recovery of epidermal barrier function may lag behind the resolution of inflammation. Taken together, these results suggest that NAP may primarily alleviate local inflammation and pruritus in AD lesions, rather than acting through systemic immunomodulation or direct barrier restoration.

### NAP treatment had a significant effect on the skin-residing microbial community

**Fig. 2:**
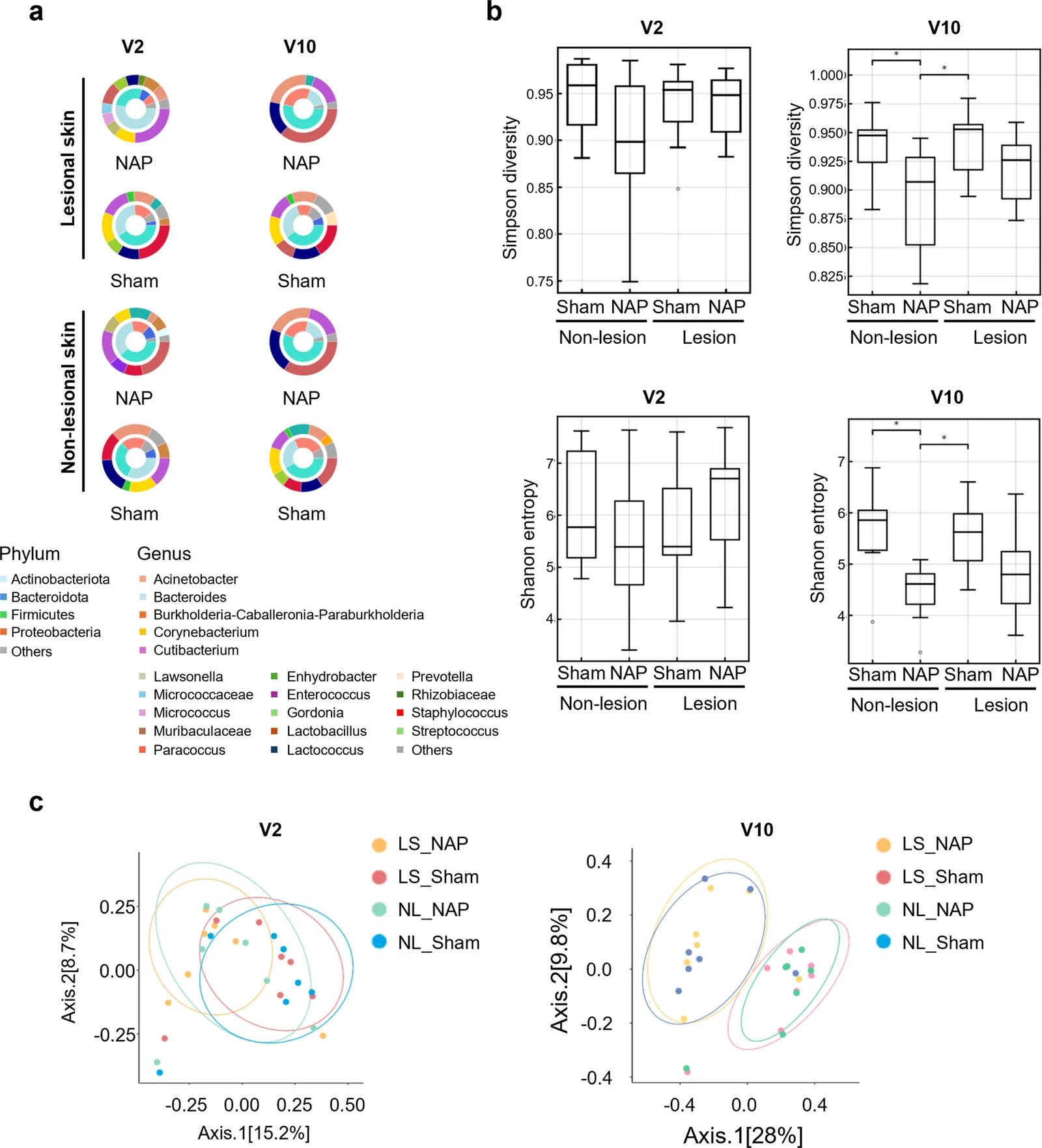
NAP treatment altered the skin microbial community, with significant changes in non-lesional skin but not lesional skin. **a** 16 S rRNA sequencing analysis of the relative abundance of major bacteria phyla and genera in lesional and non-lesional skin treated with NAP or the sham device. **b** Alpha diversity (Simpson diversity and Shannon entropy) in lesional and non-lesional skin of NAP/sham device-treated skin before (V2) and after (V10) treatment. Data are presented as box-and-whisker plots. **c** Principal coordinates analysis (PCoA) showing β-diversity of the skin microbiome based on the Jaccard distance metric in lesional and non-lesional skin before (V2) and after treatment (V10). *P* < 0.05. NAP, non-thermal argon plasma; LS, lesional skin; NL, non-lesional skin

To further investigate the mechanisms by which NAP alleviates AD lesions, we analyzed the microbiome of lesional and non-lesional skin from patients treated with NAP or the sham device using 16 S rRNA sequencing. NAP treatment significantly altered the composition of the skin microbiome, increasing the relative abundance of several genera, including *Burkholderia-Caballeronia-Paraburkholderia* and *Lactobacillus,* while decreasing *Staphylococcus*, a bacterial genus associated with AD (Fig. 2a). At V10, NAP treatment was associated with decreased α-diversity, which reflects microbial diversity within individual samples; this decrease reached statistical significance only in non-lesional skin (Fig. 2b). Notably, at V10, the microbial composition of NAP-treated skin was similar regardless of lesion status. Consistent with these findings, β-diversity, which reflects differences in microbial composition between samples, showed clustering primarily according to treatment group rather than lesion status (Fig. 2c and Fig. S3). These findings indicate that NAP treatment is associated with substantial alterations in the skin microbiome but these changes do not readily explain the specific clinical improvement observed in acute AD lesions. Nevertheless, given the reduction in *Staphylococcus*, a genus implicated in AD pathogenesis, we first asked whether this compositional shift reflects a differential susceptibility of skin bacteria to NAP.

### *Staphylococcus aureus *is more susceptible than* Lactobacillus plantarum* to the bactericidal effect of liquid plasma

**Fig. 3:**
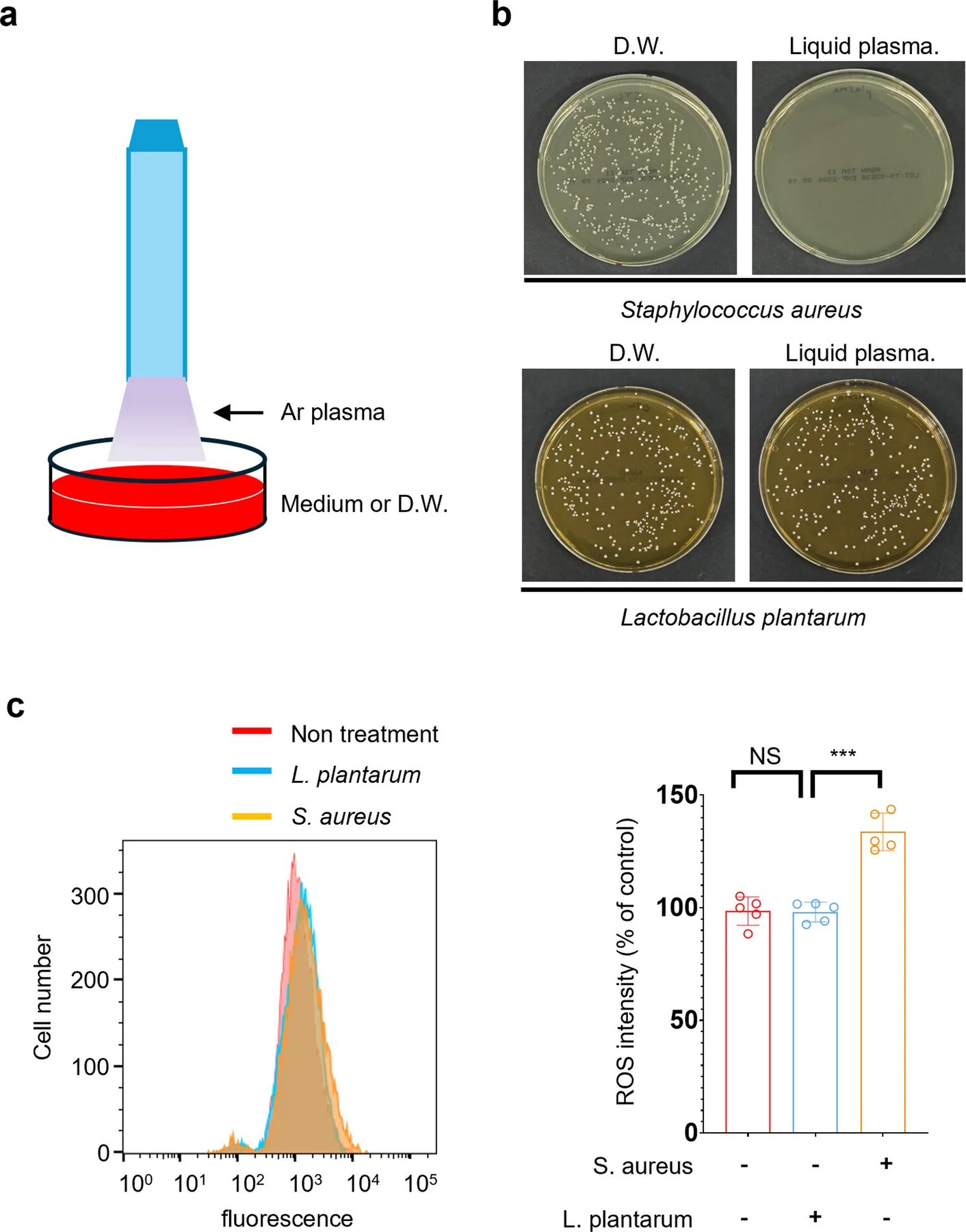
*Staphylococcus* is more susceptible than *Lactobacillus* to the bactericidal effect of liquid plasma. **a** Generation of liquid plasma. Liquid plasma was generated by treating culture medium or D.W. with NAP for 2 min/ml. **b** Bacteria (1 × 10^3^ CFU) were incubated with D.W. or liquid plasma for 10 min, spread onto culture plates, and incubated for 24 h. **c** ROS assay in HaCaT cells after bacterial co-culture (MOI = 10, *n* = 5). Data are presented as mean ± SEM. ***P < 0.001. ROS, reactive oxygen species; SEM, standard error of the mean; NAP, non-thermal argon plasma; CFU, colony-forming units; MOI, multiplicity of infection; D.W., distilled water

Given that NAP treatment increased *Lactobacillus* and reduced *Staphylococcus* in the human skin (Fig. 2a), we examined whether *Staphylococcus* was more susceptible than *Lactobacillus* to NAP. For the in vitro experiments, NAP-treated water (liquid plasma) was generated (Fig. 3a). Incubation with liquid plasma completely eradicated *S. aureus*, whereas *L. plantarum* was more resistant (Fig. 3b). These findings suggest that the reduction in *Staphylococcus* and increase in *Lactobacillus* observed in human skin after NAP treatment may result from their differential susceptibility to NAP. We next investigated whether this shift in bacterial composition affected keratinocytes. Co-culture with *S. aureus* increased reactive oxygen species (ROS) levels in HaCaT cells, whereas co-culture with *L. plantarum* had no such effect (Fig. 3c). The NAP-associated reduction in *Staphylococcus* may therefore contribute to clinical improvement by attenuating ROS production and the resulting oxidative stress in keratinocytes.

### NAP treatment ameliorated HDM-induced AD in mice

**Fig. 4:**
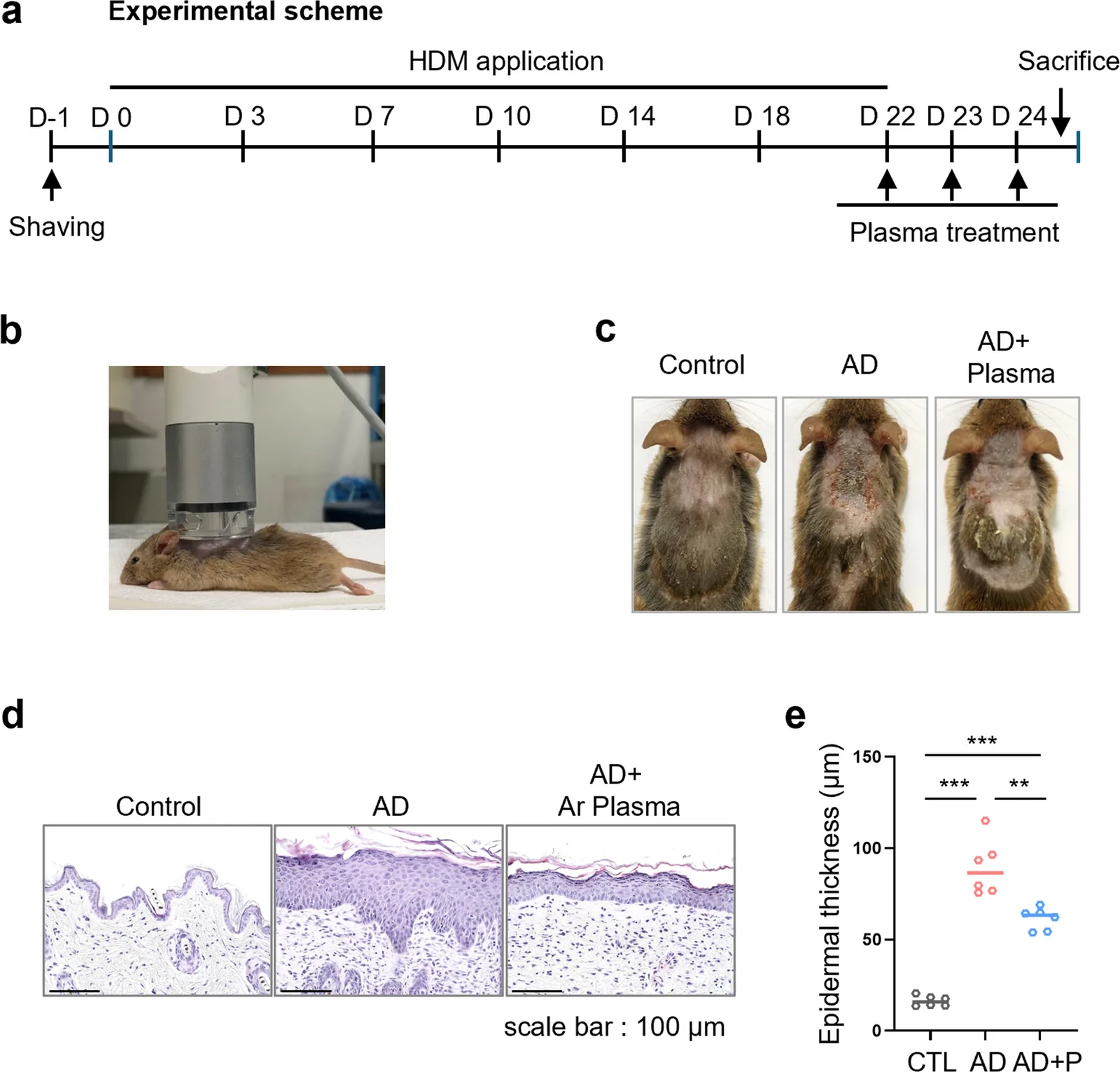
NAP treatment ameliorated HDM-induced AD in mice. **a** Experimental scheme of the AD mouse model and NAP treatment. **b** Photograph of NAP treatment on the shaved back of a mouse. **c** Representative images of AD-induced and NAP-treated mice. **d** Representative hematoxylin and eosin (H&E)-stained skin sections. Scale bar, 100 μm. **e** Epidermal thickness in the three groups (*n* = 6). Data are presented as mean ± SD. **P < 0.01; ***P < 0.001. HDM, house dust mite; AD, atopic dermatitis; NAP, non-thermal argon plasma; CTL, control; SD, standard deviation

Previous studies have demonstrated an increase in microbial α-diversity in patients whose AD lesions improved clinically [13, 14]. Because our findings differed from these reports, showing a decrease in α-diversity and a microbial shift occurring in both lesional and non-lesional skin, differential bactericidal activity alone seemed unlikely to fully account for the NAP-induced lesion-specific improvement. We hypothesized that NAP may exert keratinocyte-modulating effects that contribute to clinical improvement independently of microbiome alterations. To test this hypothesis, we induced AD in NC/Nga mice by topical application of house dust mite (HDM) extract. Following HDM application, the mice were treated with an NAP device to investigate the effects of argon (Ar) plasma treatment (Fig. 4a). Representative images of the NAP treatment are shown in Fig. 4b. NAP treatment markedly reduced the extent of AD-like lesions (Fig. 4c). Consistent with our previous study using non-thermal nitrogen plasma [15], NAP treatment inhibited immune cell recruitment (Fig. 4d), particularly that of mast cells (Fig. S4a, b) and eosinophils (Fig. S4c, d). More importantly, NAP treatment markedly attenuated epidermal acanthosis, a characteristic histopathological feature of AD (Fig. 4d, e).

### Plasma treatment suppressed allergic inflammation in AD-induced mouse skin

**Fig. 5:**
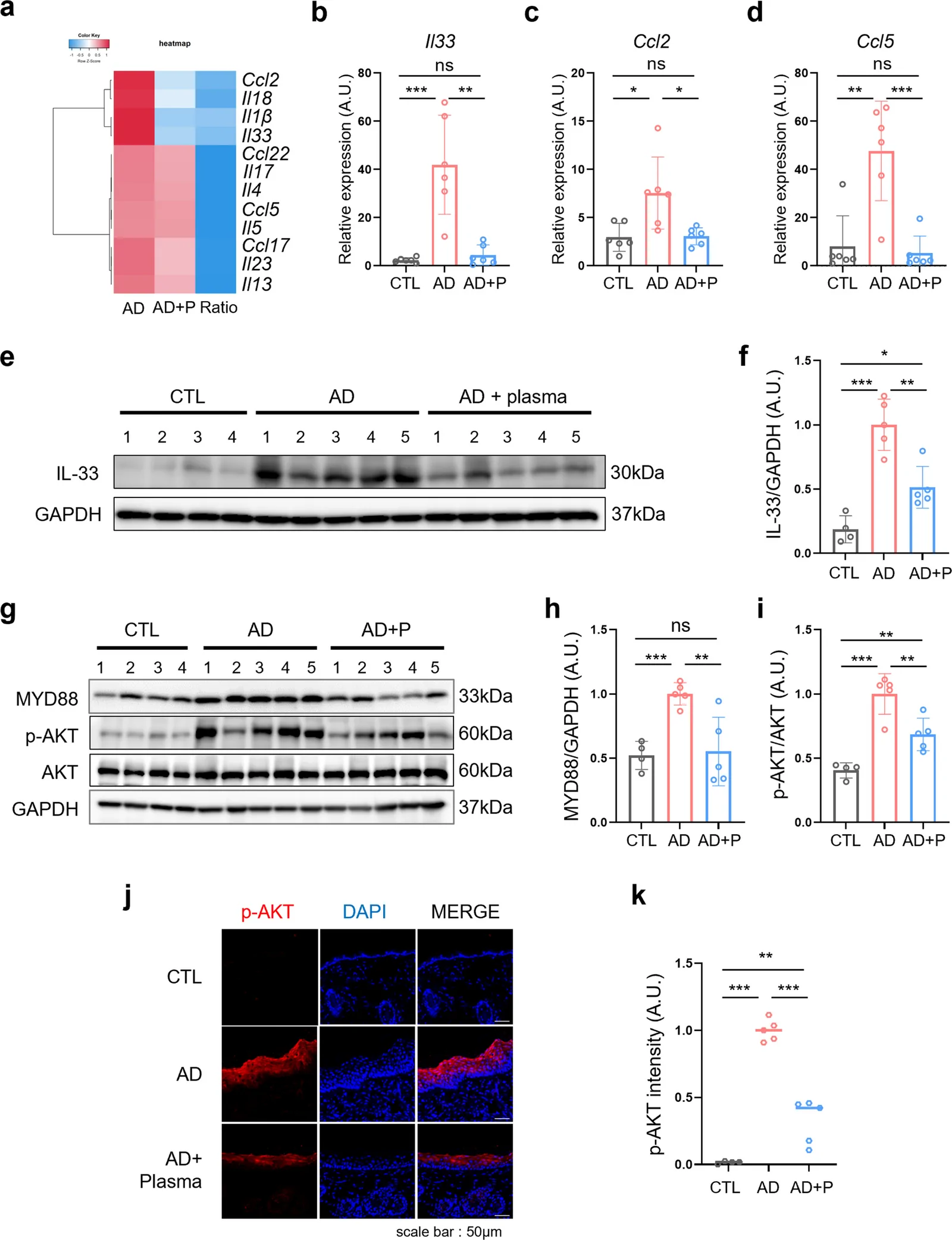
Plasma treatment suppressed allergic inflammation in AD-induced mouse skin. **a** Hierarchical clustering heat map of differentially expressed genes. The AD + P Ratio represents relative gene expression in plasma-treated AD skin (AD + P) compared with the untreated AD skin (AD). **b**–**d** Bar graphs showing relative mRNA expression of *Il33*, *Ccl2*, and *Ccl5*. **e**, **f** Representative western blot image and quantification of IL-33 protein levels in AD-induced mouse skin and after plasma treatment. **g** Representative western blot results of downstream molecules in IL-33 signaling pathway. **h**, **i** Quantification of downstream protein levels following plasma treatment. **j**, **k** Representative fluorescence microscopy images and quantification of p-AKT in control (CTL), AD, and plasma-treated AD (AD + P). Data are presented as mean ± SD. **a**–**d** *n* = 6 per group; **e**–**k** CTL, *n* = 4; AD and AD + P, *n* = 5. *P < 0.05; **P < 0.01; ***P < 0.001. CTL, control; AD, atopic dermatitis; P, plasma treatment; A.U., arbitrary units; DAPI, 4′,6-diamidino-2-phenylindole

To explore the molecular changes underlying the keratinocyte-modulating effects of NAP, we performed RNA-sequencing (RNA-seq) of AD-induced mouse skin. Several cytokines, including *Il33* and chemokines were significantly downregulated following NAP treatment (Fig. 5a). IL-33 is a major alarmin involved in various T helper 2 (Th2)-mediated diseases, including AD [16, 17]. Abrogation of IL-33 signaling decreases the expression of multiple cytokines and chemokines associated with AD [17]. Thus, we hypothesized that NAP-mediated regulation of IL-33 may help explain the observed clinical improvement. Real-time polymerase chain reaction (PCR) results also indicated that NAP inhibited *Il33* transcription (Fig. 5b). We also investigated C–C motif chemokine ligand 2/5 (*Ccl2* and *Ccl5),* chemokines associated with IL-33 signaling that are important for the recruitment of effector cells during allergic responses [18–20]. As expected, *Ccl2* and *Ccl5* expression increased in HDM-induced AD skin, whereas NAP treatment significantly reduced their transcription (*P* < 0.05 and *P* < 0.001, respectively; Fig. 5c, d). IL-33 protein levels were consistent with the mRNA finding, increasing in AD skin compared with controls and decreasing significantly after NAP treatment (Fig. 5e, f). We further examined the IL-33 signaling pathway by western blotting. Myeloid differentiation primary response protein 88 (MYD88) and phosphorylated AKT (p-AKT) levels increased in AD-induced mouse skin and were reduced by NAP treatment (Fig. 5g–i). This confirmed the IL-33 dependent signaling process in mouse skin, which was capable of being attenuated by NAP. Immunofluorescence microscopy confirmed increased p-AKT intensity in AD lesions, which was significantly reduced after NAP treatment (Fig. 5j, k). These findings suggest that NAP attenuates IL-33-associated inflammatory signaling in AD-induced mouse skin, providing a biologically plausible mechanism for its therapeutic effects.

### Plasma treatment reduced oxidative stress in AD-induced mouse skin

**Fig. 6:**
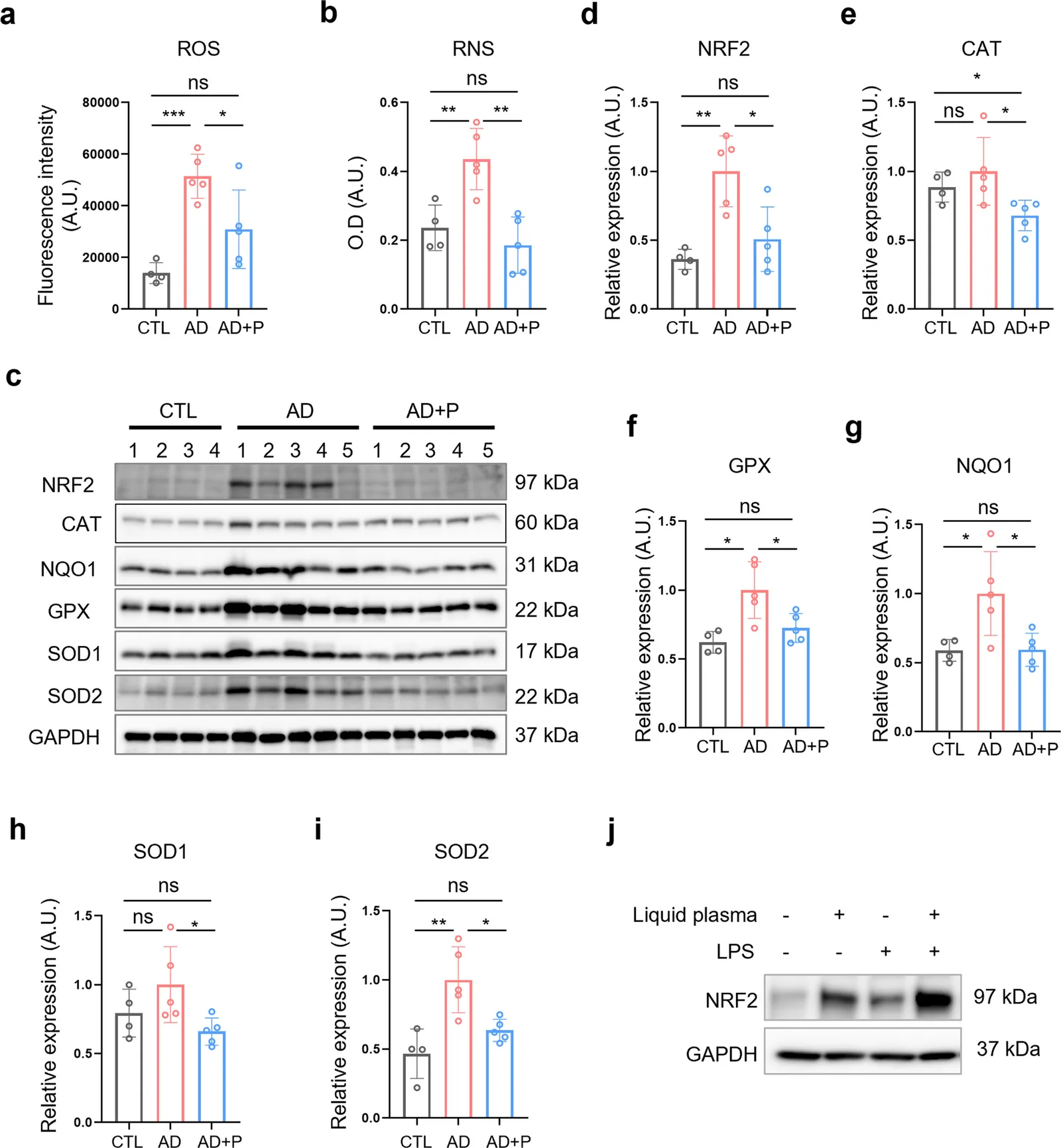
Plasma treatment reduced oxidative stress in AD-induced mouse skin. **a** Total ROS levels. **b** Total RNS levels. **c** Representative western blotting results of antioxidant molecules. **d**–**i** Quantification of antioxidant enzyme protein levels after plasma treatment. **j** Representative western blotting results for NRF2 with or without LPS stimulation or plasma treatment. Data are presented as mean ± SD. CTL, *n* = 4; AD and AD + P, *n* = 5. *P < 0.05; **P < 0.01; ***P < 0.001. ROS, reactive oxygen species; RNS, reactive nitrogen species; AD, atopic dermatitis; P, plasma treatment; CTL, control; O.D., optical density; A.U., arbitrary units; NRF2, nuclear factor erythroid 2-related factor 2; LPS, lipopolysaccharide

Because OS is known to enhance IL-33 expression and release in epithelial tissues [21, 22], we examined whether NAP treatment reduced ROS and reactive nitrogen species (RNS) levels and modulated OS-related signaling pathways in AD-induced mouse skin. Total ROS levels were increased in AD-induced mouse skin, and NAP treatment suppressed this increase (Fig. 6a). RNS levels showed a similar pattern, increasing in AD mice and decreasing after plasma treatment (Fig. 6b). We also measured nuclear factor erythroid 2-related factor 2 (NRF2) and antioxidant proteins, including catalase (CAT), glutathione peroxidase (GPX), NAD(P)H:quinone oxidoreductase 1 (NQO1), and superoxide dismutase 1 (SOD1) and 2 (SOD2). NRF2 and antioxidant protein expression were increased in AD-induced mouse skin and were attenuated by NAP treatment (Fig. 6c–i). The increased expression of antioxidant enzymes likely represents a defense mechanism against elevated OS. However, this response was insufficient to counteract the increased ROS levels in AD-induced mouse skin, as evidenced by AD-like symptoms.

**Fig. 7:**
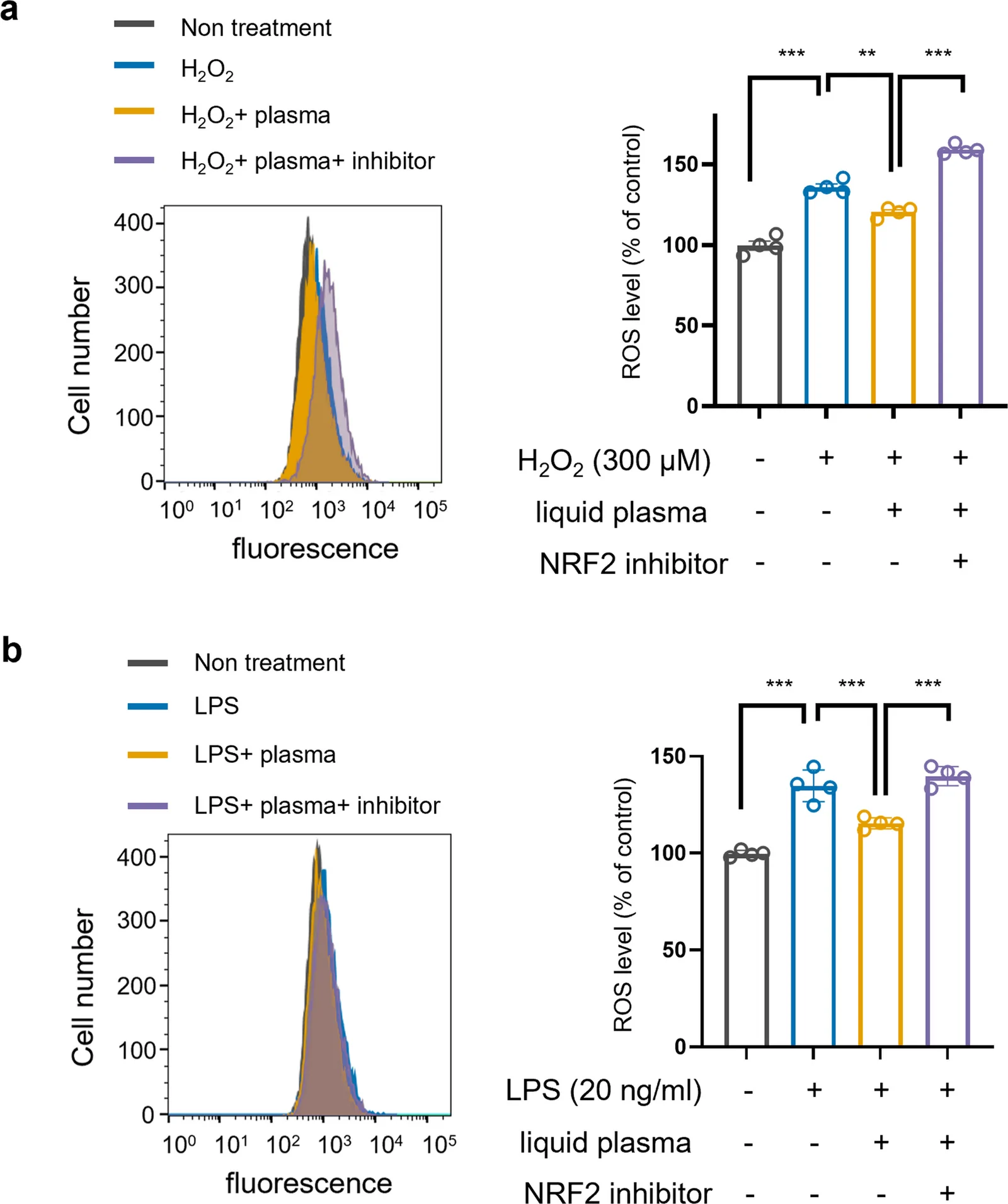
Antioxidant activity of liquid plasma is via NRF2 pathway. HaCaT cells were stimulated with (**a**) H_2_O_2_ or (**b**) LPS and ROS levels were determined by flow cytometry. Data are expressed as means ± SEM. (*n* = 4) **P < 0.01, ***P < 0.001. LPS, lipopolysaccharide; ROS, reactive oxygen species; NRF2, nuclear factor erythroid 2-related factor 2; SEM, standard error of the mean

We therefore reasoned that NRF2 activation by NAP treatment might increase antioxidant protein expression sufficiently to control the ROS increase. To test this hypothesis, we stimulated HaCaT cells, a human keratinocyte cell line, with lipopolysaccharide (LPS) and treated them with liquid plasma generated by NAP. LPS stimulation induced NRF2 expression, and plasma treatment further increased NRF2 expression (Fig. 6j). In addition, treatment of HaCaT cells with an NRF2 inhibitor diminished the antioxidant activity of liquid plasma (Fig. 7a, b). These findings support the involvement of the NRF2 pathway in the antioxidant activity of plasma treatment. Taken together, our results suggest that plasma treatment enhances antioxidant defense and may thereby counteract elevated OS and alleviate AD-like symptoms.

## Discussion

**Fig. 8:**
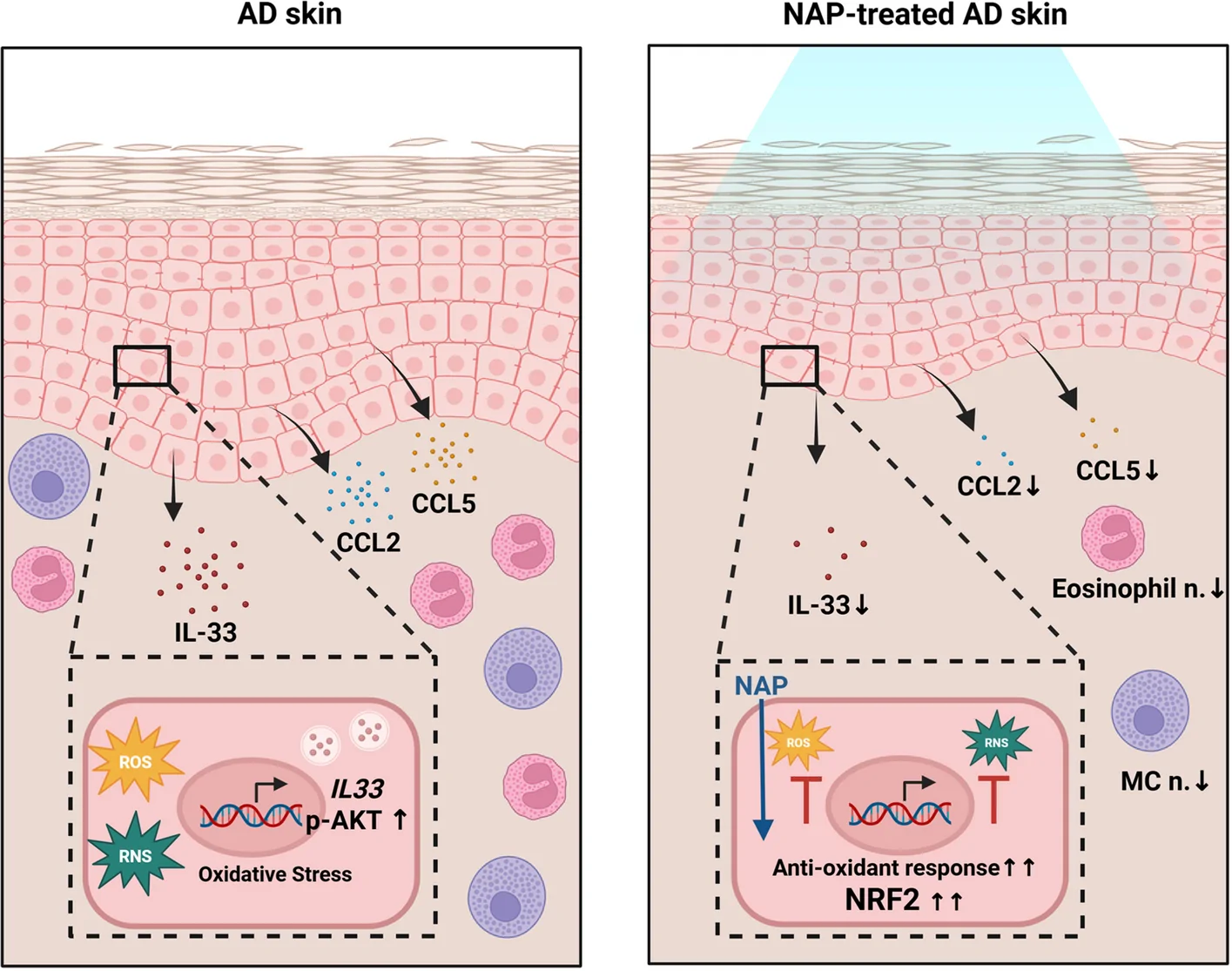
Schematic representation of atopic dermatitis inhibition via plasma treatment. NAP may alleviate AD by enhancing NRF2-associated antioxidant responses, reducing oxidative stress, and attenuating IL-33-associated inflammatory signaling and inflammatory cell infiltration. Created in BioRender. Park, Y. J. (2026) https://BioRender.com/13p0fus. BioRender Academic Publication License agreement number: QB2A9LXLJW. AD, atopic dermatitis; NAP, non-thermal argon plasma; NRF2, nuclear factor erythroid 2-related factor 2; IL-33, interleukin-33; CCL2, C–C motif chemokine ligand 2; CCL5, C–C motif chemokine ligand 5; MC, mast cell; OS, oxidative stress; RNS, reactive nitrogen species; p-AKT, phosphorylated AKT

In this study, we demonstrated that NAP improved acute eczematous lesions in patients with AD. Using an AD mouse model, we also investigated the potential mechanisms underlying the therapeutic effects of NAP and found that NAP may attenuate IL-33 expression and oxidative stress in keratinocytes independently of alterations in the skin microbiome (Fig. 8).

Our human skin microbiome analysis revealed decreased α-diversity in NAP-treated areas accompanied by symptom improvement. This finding differs from previous studies reporting increased α-diversity upon clinical improvement. This discrepancy may highlight the complexity of the relationship between microbiome composition and AD severity. In addition, a previous study demonstrated that dysbiosis can precede the onset of AD [23], suggesting that not all microbiome alterations necessarily contribute to disease worsening. Therefore, the microbiome alterations observed in this study should be interpreted as an associated finding rather than direct evidence of a causal mechanism underlying the therapeutic effects of NAP.

Multiple studies have underscored the role of OS in AD [24]. However, the regulation of OS in keratinocytes is complex, and few studies have directly examined its association with clinical improvement. Quist et al. investigated the effect of topical agents (tacrolimus 0.1% ointment vs. polyunsaturated fatty acid-containing lotion) on OS in patients with AD [25]. OS markers measured by gas chromatography–mass spectrometry in the interstitial fluid collected in vivo by dermal microdialysis showed that tacrolimus ointment reduced OS. Our findings are consistent with previous reports of increased OS in patients with AD and suggest that alleviation of OS may contribute to clinical improvement.

IL-33 is a cytokine associated with inflammatory responses. IL-33 dysregulation has been implicated in the pathogenesis of several Th2-mediated diseases, including asthma, allergic conjunctivitis, and allergic rhinitis. Patients with AD also exhibit increased IL-33 levels in keratinocytes and monoclonal antibodies targeting IL-33 or the IL-33 receptor (IL-33R) have reached clinical development [26]. However, recent studies have shown that IL-33 targeting antibodies do not necessarily produce significant clinical improvement in patients with chronic or severe AD, in contrast to some other allergic diseases [27]. Chronic AD differs from acute AD not only in clinical manifestations but also in persistent immune dysregulation, epidermal remodeling, and a more complex inflammatory milieu, suggesting that pathogenic pathways beyond IL-33 contribute to disease persistence [28]. Therefore, the findings of the present study, which included only patients with mild-to-moderate AD presenting predominantly with acute eczematous lesions, should not be directly extrapolated to chronic or severe AD. Instead, our data suggest that acute lesions may be more responsive to modulation of the IL-33 pathway and that NAP may influence IL-33 signaling to an extent that contributes to clinical improvement. Whether NAP exerts comparable therapeutic effects in patients with chronic AD remains to be determined in future clinical studies.

Our study has several limitations that warrant further investigation. First, the exploratory nature of the study and small sample size limit the generalizability of the findings. Nevertheless, no serious adverse events were observed throughout the study, supporting a favorable safety profile of NAP within the limitations of this exploratory trial. Second, although we identified microbiome alterations, we did not perform species-level analysis using whole-metagenome sequencing. This limitation prevented functional annotation and detailed assessment of the effects of NAP treatment on the skin microbiome. The swab specimens did not contain sufficient bacterial DNA necessary for whole-metagenome sequencing, which precluded a more detailed analysis. Third, although our findings support a role of NAP in alleviating OS through modulation of NRF2 expression, we did not determine whether NAP reduces OS in human skin or assess its effects on other cellular components of human skin in vivo. Thus, although our study provides a plausible mechanistic explanation, it does not establish this mechanism in human AD. Mechanistic findings from murine and in vitro models should therefore be interpreted as exploratory and biologically supportive rather than direct evidence of the mechanism underlying clinical efficacy in humans. Fourth, the clinical component of this study was retrospectively registered after completion of participant follow-up, as prospective registration was not completed because of an administrative oversight at study initiation. Retrospective registration may introduce selective reporting bias and affect the reliability of the clinical findings. However, the clinical outcomes (EASI, IGA, NRS, and DLQI) were prespecified in the IRB-approved protocol before enrollment, and all prespecified outcomes are reported in this manuscript. Nevertheless, these findings should be interpreted with caution and confirmed in prospectively registered trials. Comprehensive investigation of the interactions between NAP and other elements involved in atopic dermatitis remains an area for future research. Future studies should also evaluate the effects of NAP on chronic lesions and include long-term follow-up to assess sustained efficacy and safety. Broader safety assessments, such as skin histopathology before and after treatment, and head-to-head comparisons with conventional AD therapies, including topical corticosteroids, would also be valuable.

Despite these limitations, this exploratory study suggests that NAP may improve mild-to-moderate AD, particularly acute eczematous lesions, and represent a promising non-pharmacological treatment option. Findings from the murine and in vitro models further support the potential involvement of NRF2-associated antioxidant responses and attenuation of IL-33 signaling in its therapeutic effects.

## Materials and methods

### Clinical trial design

This was a double-blind, randomized, sham, device-controlled, parallel-design, phase I study of adults with mild-to-moderate AD. Fifteen patients were enrolled and randomized in a 2:1 ratio into the NAP (*n* = 10) and sham (*n* = 5) groups using a random-number table. To maintain double blinding, the sham device was matched to the active NAP device of appearance, operating sound, and treatment sensation but did not generate therapeutic plasma, thereby preventing participants and outcome assessors from distinguishing between the two interventions. The Study was conducted at Ajou University Hospital between September 2021 and January 2023. The clinical trial consisted of a screening period (days 14 to 1), followed by a 7-week double-blind treatment period, during which patients received NAP treatment twice a week for 4 weeks (a schematic summary of the study design is shown in Fig. S1a). For safety monitoring, patients were followed up for 2 weeks after the end of treatment (V11), during which no treatment was administered.

### Participants

Eligible patients aged 18–75 years, had investigator-assessed mild-to-moderate AD, had a documented history of AD treatment, and were willing to discontinue oral medications and topical agents during NAP treatment. Patients were defined as having mild AD if they had both investigator global assessment (IGA; 5-point severity scale from clear to severe, wherein 0 is clear, 1 is almost clear, 2 is mild, 3 is moderate, 4 is severe) of two and eczema area severity index (EASI) score of 1.1 ≤ EASI ≤ 7.0 at screening and baseline, whereas moderate AD was defined as IGA score of 3 and EASI score of 7.1 ≤ EASI ≤ 21.0. Concomitant use of any treatments other than topical emollients was prohibited. Detailed eligibility criteria are provided in Appendix S1. Rescue medication consisted of topical mometasone furoate ointment administered at the discretion of the investigator, a board-certified dermatologist. Patients provided written informed consent for publication of their case details.

### Physical properties of NAP device and treatments

Argon gas (Ar) was used as the working gas for non-thermal atmospheric plasma generation. The gas flow rate was 5 L/min, the input voltage was 15 kV, and the frequency was maintained at 15 kHz. Fig. S1b shows the exterior of the Ar-based NAP device used for AD treatment. The properties of the Ar-based NAP device are shown in Fig. S1c, d. Briefly, optical emission spectroscopy was performed over a wavelength range of 280–920 nm using an optical emission spectrometer (SV2011, K-MAC, Daejeon, Republic of Korea) to identify the particles and radicals generated by the Ar plasma system. Argon ions dominated the plasma emission spectrum. Five lesions were irradiated for 1 min at each visit. Each patient received nine treatments over 4 weeks.

### Efficacy and safety assessments

To evaluate the efficacy and safety of NAP in adults with AD, the primary endpoint was the change in EASI and IGA scores from baseline to V10. These scores were evaluated by both the investigator and an independent evaluator at 2 and 4 weeks (V6 and V10) after the first NAP application. Secondary endpoints included changes from baseline in the pruritus Numeric Rating Scale (NRS), Dermatology Life Quality Index (DLQI), and transepidermal water loss (TEWL). Safety and tolerability were assessed by the incidence of treatment-emergent adverse events (TEAEs), vital signs, laboratory parameters, and electrocardiograms throughout the study.

### 16 s rDNA gene sequencing

A DNeasy PowerSoil Pro Kit (QIAGEN, Hilden, Germany) was used to extract genomic DNA from the samples. DNA concentration was measured using a NanoDrop and Qubit (Thermo Fisher Scientific, Waltham, MA, USA). The V3-V4 hypervariable regions of the 16 s rRNA gene were amplified by PCR using fusion primers. The sequencing library was constructed by attaching a multiplexing index and an Illumina sequencing adapter to the PCR products according to the Illumina protocol. The final product, including the Illumina adapter and multiplexing index, was approximately < 630 bp in size. Paired-end reads (2 × 300 bp) were generated using MiSeq platform (Illumina, USA).

### Microbiome data analysis

Illumina adapter sequences were removed using Cutadapt (version 3.2). DADA2 (version 1.20) and QIIME 2 (version 2011.11) were used to eliminate quality trimming of the phix sequences. Error rate calculations, merging of paired ends, and de-chimerization were performed using DADA2. Taxonomy was assigned to the resulting amplicon sequence variant table using the SILVA database (version 138). Rarefaction was performed to confirm sufficient sequencing depth for downstream analysis. Taxonomy of the rarefied amplicon sequence variant table was assigned from the phylum to genus level, and relative abundance was calculated. Only taxa with a relative abundance and prevalence > 10% in each group were included in the analysis. Alpha and beta diversities were calculated to analyze the diversity between and within samples. Statistical analyses were performed using R scripts and the phyloSeq package. The Wilcoxon rank-sum test and nonparametric statistical analysis were used to verify the significance of alpha diversity. Beta diversity was evaluated using Jaccard distance, and statistical significance was assessed by permutational multivariate analysis of variance (PERMANOVA). Results were visualized using matplotlib and ggplot2 packages.

### Cells, bacteria and reagents

HaCaT cells were cultured in Dulbecco’s modified Eagle medium (DMEM) (Welgene, Daegu, Korea) supplemented with 10% fetal bovine serum (Gibco, Waltham, MA, USA) and 1% Antibiotic–Antimycotic (Gibco, 15,240–062). Cells were incubated at 37 °C in 5% CO_2_ under humidified conditions. *Staphylococcus aureus* (ATCC 6538) and *Lactobacillus plantarum* were purchased from the American Type Culture Collection (Manassas, VA, USA) and the Korean Collection for Type Cultures (South Korea), respectively. For the disinfection assay, bacteria were incubated in distilled water (D.W. or liquid plasma for 10 min, spread onto bacterial culture plates, and incubated overnight. Cells were authenticated by short tandem repeat analysis and mycoplasma contamination detection test was conducted to confirm mycoplasma-free.

### ROS assay

The ROS assay with cells was performed using dihydroethidium (Invitrogen Carlsbad, CA, USA). HaCaT cells were seeded in 12-well plates at a density of 5 × 10^4^ cells/well and incubated for 24 h with or without liquid plasma. The following day, cells were treated with dihydroethidium and incubated for 30 min at 37 °C in 5% CO_2_. Bacteria, LPS, or 300 µM H_2_O_2_ was added to induce ROS production, followed by incubated for 1 h. After ROS induction, cells were detached and analyzed by flow cytometry.

ROS levels in mouse skin were determined using a hydrogen peroxide assay kit (ab102500, Abcam, Melbourne, Australia) according to the manufacturer’s instructions. Nitric oxide concentrations in the mouse skin under the experimental conditions were measured using an NO assay kit (ab65328, Abcam, Melbourne, Australia). Samples were prepared and analyzed according to the manufacturer’s instructions.

### Mice and animal experiments

Six-week-old, male NC/Nga mice were purchased from Orient Bio (Seoul, South Korea). The mice were housed in a controlled room under a 12:12 h light–dark cycle and free access to laboratory chow and water. Eight-week-old NC/Nga mice were used for the animal experiments. The animal experiments were approved by the Committee for Ethics in Animal Experiments of Ajou University School of Medicine and performed in accordance with institutional guidelines (approval number: 2017–0026).

AD was induced in NC/Nga mice by applying a house dust mite (HDM)-containing cream (Biostir, Japan), as described previously, with minor modifications [12]. Briefly, dorsal hair was completely removed from each mouse. The following day, 200 µL of 4% sodium dodecyl sulfate (SDS) solution was applied to the dorsal skin to disrupt the skin barrier. After 6 h, 100 mg Biostir cream was applied twice weekly for 3 weeks. AD-induced mice were divided into three groups: non-treated control group (*n* = 4), Biostir-treated control group (*n* = 5), and a Biostir-treated group receiving topical application of plasma (*n* = 5). Plasma treatment was administered on days 21, 22, and 23 for 2 min/day for 3 consecutive days. Mice were sacrificed 6 h after the final treatment.

### RNA isolation, cDNA synthesis and real-time PCR

RNA was isolated from mouse skin tissue using TRIzol reagent (Gibco, Carlsbad, CA, USA). Subsequently, first-strand of cDNA was synthesized from 1 μg total RNA using a ReverTra Ace qPCR RT Master Mix (TOYOBO, Japan). Primer sets for *Il33*, *Ccl2*, *Ccl5*, and glyceraldehyde −3-phosphate dehydrogenase (*Gapdh*) were purchased from Qiagen (Hilden, Germany). *Gapdh* transcript levels were used for normalization. The amplification program consisted of one cycle at 95 °C for 10 min, followed by 45 cycles at 95 °C for 20 s, 55 °C for 20 s, and 72 °C for 20 s.

### Western blot analysis

Western blotting was performed as previously described, with minor modifications [12]. Cells were lysed using radioimmunoprecipitation assay (RIPA) buffer (Sigma-Aldrich, St. Louis, MO, USA) containing a protease inhibitor cocktail and PhosSTOP (Roche Molecular Biochemicals, Basel, Switzerland). Ten micrograms of protein lysate were subjected to sodium dodecyl sulfate–polyacrylamide gel electrophoresis (SDS-PAGE) and transferred to polyvinylidene fluoride (PVDF) membranes (Amersham, Arlington Heights, IL, USA). After transfer, the membranes were incubated with a blocking solution, and subsequently with the appropriate antibodies. Bands were visualized using advanced enhanced chemiluminescence (ECL) western blot detection reagents (Amersham). GAPDH was used as a loading control.

### Histological analysis

Dorsal skin was fixed in 4% paraformaldehyde for 24 h and embedded in paraffin. Sections (5 μm) were prepared and stained with hematoxylin and eosin to assess histological changes in the skin. Slides were reviewed by a dermatologist (HSK) and a trained dermatopathologist (YJP).

### Statistical analysis

Data are presented as the mean ± standard deviation (SD) or standard error of the mean (SEM), as indicated in the corresponding figure legends. Comparisons between the two groups were performed using an unpaired, two-tailed Student's *t*-tests. Comparisons among the three groups were performed using one-way analysis of variance (ANOVA), followed by Tukey’s multiple-comparison test. Differences were considered statistically significant at ^*^P < 0.05, ^**^P < 0.01, and ^***^P < 0.001. All statistical analyses were performed using GraphPad Prism version 10.6.1.

## Supplementary Information

Supplementary Material 1.

## Data Availability

All data generated or analyzed during this study are included in this published article and its supplementary files. The RNA-sequencing data generated in this study are publicly available in the Figshare repository at https://doi.org/10.6084/m9.figshare.34337121. Additional datasets, including the 16S rRNA sequencing data, are available from the corresponding authors upon reasonable request.
