# Supplementary material for: Non-thermal argon plasma therapy alleviates atopic dermatitis by reducing keratinocyte oxidative stress
Source: Mol Biomed. 2026 Nov 2;7:236. doi: 10.1186/s43556-026-00641-y (PMC13728056; doi:10.1186/s43556-026-00641-y)
Supplement: Supplementary file 1 [file 43556_2026_641_MOESM1_ESM.docx]

**Supplementary information**

**Non-thermal argon plasma therapy alleviates atopic dermatitis by reducing keratinocyte oxidative stress**

Young Joon Park, MD, PhD^a,^ ^†^, Mei Huang^d, †^, Yun Sang Lee, PhD^c, †^, Han-Seul Kim, MD^a^, Hee Young Kang, MD, PhD^a,*^, Chul-Ho Kim, MD, PhD^b,c,*^

*^a^Department of Dermatology, Ajou University School of Medicine, Suwon, Korea*

*^b^Department of Molecular Science and Technology, Ajou University School of Medicine, Suwon, Korea*

*^c^Department of Otolaryngology, Ajou University Medical Center, Suwon, Korea*

*^d^Department of Medical Sciences, Graduate School of Ajou University, Suwon, Korea*

†These authors contributed equally to this work.

**^#^Corresponding authors:**

Hee Young Kang

Department of Dermatology, Ajou University School of Medicine, 164 WorldCup-ro, Yeongtong-gu, Suwon, 16499, Korea

Tel: +82-31-219-5188

Fax: +82-31-219-5189

E-mail: hykang@ajou.ac.kr

Chul-Ho Kim

Department of Otolaryngology, Ajou University School of Medicine, 164 WorldCup-ro, Yeongtong-gu, Suwon, 16499, Korea

Tel: +82-31-219-5269

Fax: +82-31-219-5264

E-mail: ostium@ajou.ac.kr

**Appendix S1. Full patient eligibility criteria in our pilot study**

- Inclusion criteria

1. Men and women ages between 19 and 75 years old.

2. Diagnosed with atopic dermatitis according to Korean diagnostic criteria for atopy (at least two primary diagnostic criteria, and at least four secondary diagnostic criteria)

3. Atopic dermatitis persisting for at least 24 weeks with more than 5% total body surface area (BSA)

4. Atopic dermatitis classified as mild (Investigator’s global assessment (IGA) score of 2 and Eczema Area and Severity Index (EASI) score between 1.1 and 7.0) or moderate (IGA score of 3 and EASI score between 7.1 and 21.0) at screening and baseline visit.

5. Patients willing to participate in the trial, comply with treatment and procedures, and visit the trial institution for all observational evaluations.

6. Patients who have listened to explanations about the purpose and method of this clinical trial and voluntarily signed a written consent form.

- Exclusion criteria

Participants meeting any of the following conditions cannot participate in this clinical trial:

1. Atopic dermatitis involving the periocular or genital area

2. Patients with medical history or comorbid conditions that may affect the results.

A. Serious skin diseases other than atopic dermatitis (e.g., T-cell lymphoma, allergic contact dermatitis, recurrent herpes, active skin infection, etc.) or extensive scars in the area of atopic dermatitis lesions.
B. Active itching due to chronic urticaria, allergic substances, insect bites, etc.
C. Uncontrolled hypertension
   : systolic blood pressure >160 mmHg or diastolic blood pressure >100 mmHg.
D. Uncontrolled diabetes: HbA1c≥ 8.0%.
E. Severe asthma or uncontrolled asthma:

: asthma worsening requiring systemic steroids for more than 3 days occurs more than twice a year.

F. Liver disease or liver dysfunction: AST or ALT > 5 times the upper limit of normal.
G. Severe kidney disease: serum creatinine > 3 times the upper limit of normal.
H. Bleeding tendency: PT and aPTT > 1.5 times the upper limit of normal,
I. Other severe comorbidities that the investigator deems inappropriate for participation in the clinical trial (e.g.; heart disease, kidney disease, liver disease, respiratory disease, malignant tumors, immunodeficiency, etc.).

3. Patients with therapeutic interventions that could potentially interrupt this clinical trial

A. Received systemic therapy with steroids, immunosuppressants, or antibiotics, or undergone photochemical therapy within 28 days prior to the first application of the investigational medical device for the clinical trial.
B. Received topical therapy for atopic dermatitis within 14 days prior to the first application of the investigational medical device for the clinical trial, involving the use of steroids, immunosuppressants, or antibiotics for the purpose of treating atopic dermatitis.
C. Received light therapy procedures within 14 days prior to the first application of the investigational medical device for the clinical trial.
D. Received omalizumab or dupilumab treatment within 24 weeks prior to the first application of the investigational medical device for the clinical trial.
E. Received a cardiac pacemaker implant.

4. Patients who have participated in another interventional clinical trial, received investigational medicinal products (in case of biologics, within 16 weeks or within 5 times the half-life), or undergone procedures with investigational medical devices within 30 days prior to the screening time point.

5. Pregnant or breastfeeding female patients

6. Fertile women and men who have plans for pregnancy during the clinical trial period or do not agree to use appropriate contraception*.

* Hormonal contraceptives, intrauterine devices or systems, dual contraception (using both male condoms and barrier methods for females, such as diaphragms or cervical caps), fertility procedures, etc.

7. Individuals for whom participation in this clinical trial is deemed inappropriate based on the medical judgment of the investigator

**Supplementary table**

| **Table S1. Baseline participant characteristics** | | |
| --- | --- | --- |
|  | Sham-control group (n = 5) | Active NAP group (n = 10) |
| Age, years, mean ± SD | 35.4 ± 12.5 | 40.0 ± 19.2 |
| Sex, male, n (%) | 3 (60.0) | 7 (70.0) |
| Duration of atopic dermatitis, years, mean ± SD | 20.0 ± 10.6 | 10.3 ± 8.6 |
| Baseline EASI score, mean ± SD | 6.4 ± 3.3 | 7.6 ± 3.4 |
| Baseline pruritus NRS score, mean ± SD | 5.0 ± 1.9 | 5.9 ± 2.1 |
| Baseline DLQI score, mean ± SD | 6.0 ± 2.4 | 11.9 ± 7.3 |
| Abbreviation: DLQI, Dermatology Life Quality Index; EASI, Eczema Area and Severity Index; NAP, nonthermal argon plasma; NRS, numerical rating scale; SD, standard deviation. | | |

**Supplementary figure**

**Fig. S1. Schematic summary of our exploratory clinical trial and properties of the non-thermal argon plasma (NAP) device.** (a) Study design and timeline of the exploratory clinical trial, including the screening, randomization, treatment, and follow-up visits. A total of 15 mild to moderate AD patients participated in the trial of 6 to 10 weeks. (b) Exterior of non-thermal plasma device for AD treatment. (c) Optical emission spectra profile and (d) current-voltage profile of the NAP device. AD, atopic dermatitis; NAP, non-thermal argon plasma; V, visit.

**Fig. S2. Clinical improvement by NAP treatment is not due to skin barrier improvement or systemic suppression to allergic response.** (a) TEWL score and (b) total IgE level between the NAP-treated group and Sham device-treated group. Data are expressed as means ± SEM. NAP, non-thermal argon plasma; TEWL, transepidermal water loss; IgE, immunoglobulin E; SEM, standard error of the mean.

**Fig. S3. NAP renders the composition of bacterial community regardless of lesional and non-lesional skin.** When beta diversity is plotted by PCA, the diversity does not significantly differ between the lesion and non-lesional skin treated with NAP at the end of treatment (V10). NAP; Non-thermal argon plasma, PCA; Principal coordinate analysis; LE, lesional skin; NL, non-lesional skin; CTRL, control; V2, visit 2; V10, visit 10; 1MIN, 1-min NAP treatment.

**Fig. S4. Ar-based Non-thermal treatment inhibits infiltration of mast cells and eosinophils.** (a) Representative staining of mast cells showing plasma treatment inhibits mast cell infiltration in the mouse AD-induced skin. (b) Infiltrated mast cell numbers were indicated by the graph. (c) Eosinophil infiltration was determined by immunofluorescence microscopy. (d) The graph shows increased eosinophil infiltration in the mouse AD-induced skin, and plasma treatment inhibits the eosinophil infiltration. (n = 6) **P < 0.01, ***P < 0.001. Ar, argon; AD, atopic dermatitis; CTL, control; P, plasma treatment; HPF, high-power field.
